# Buttermilk

**Published:** 1905-06

**Authors:** 


The Business office of THE JOURNAL-RECORD is No. 1007-1008 Century Building.

The Editorial office is 318-319-320 The Prudential.

Address all Business communications to Dr. M. B. Hutchins, Mgr.

Make remittances payable to THE ATLANTA JOURNAL-RECORD OF MEDICINE.

On matters pertaining to the Editorial and Original Communications address Dr Bernard Wolff, Atlanta.

Reprints of original articles will be furnished at cost price. Requests for the same should always be made *on the manuscript*.

We will present, post-paid, on request, to each contributor of an original article, twenty (20) marked copies of THE JOURNAL-RECORD containing such article.

### BUTTERMILK.

A FRENCH exchange (*Le Proges Medical*) seeks to revive interest as a therapeutic agent in the simple and natural alimentary product, buttermilk. Buttermilk is a fluid derived from milk when butter is made in the old-fashioned way, the cream skimmed and butter gotten therefrom by churning. The fluid remaining after the butter has been obtained has on the average the following composition:

| | Grms. | Cent. |
|---|---|---|
| Lactic acid, about | 0 | 75 |
| Residual butter | 1 | 00 |
| Earthy constituents | 4 | 03 |
| Extractives | 4 | 04 |
| Ash | 0 | 72 |
| Water | 90 | 12 |

The acidity of buttermilk increases with age; the change being more rapid with high temperature, and finally stops with the maximum degree of lactic acid fermentation. It is exactly the same process that occurs in souring milk which left exposed to the air or with the addition of the ferment selected by Metchinkoff (lactobacilline). Non-acid buttermilk may be obtained by churning *fresh* cream, but it very soon takes on a bitter taste which renders it less useful. The buttermilk used is generally acid or neutral, depending upon its age, and this changing composition explains the apparent contradiction in its dietetic application. By normal buttermilk is meant that fluid slightly acid, to be used not later than twenty-four hours after it has been obtained from churning cream or slightly sour milk.

The nutritive power of buttermilk is slight, since it contains scarcely one per cent. of butter and less sugar than ordinary sweet milk. It is a *stimulant* and *antiseptic* by virtue of the lactic acid contained, and *carminative* by reason of a certain transformation, as yet unknown, of its caseine. For a long time it has been used in Holland as a food for adults, nurslings and young animals. Its therapeutic indications appear to be real, and the following are worthy of remark: In chronic atonic constipation, and in cholera infantum and gastro enteritis of children. Good results have been secured by its use in febrile states, in the vomiting of pregnancy and in Bright's Disease. For constipation it should be given in quantities from 250 to 500 c.c. early in the morning. As an artificial food for babies, the following is proposed:

| | |
|---|---|
| Wheat flour | 20 grms. |
| Powdered sugar | 50 grms. |
| Buttermilk | 1 litre. |

Add the flour to a little of the buttermilk cold, then sweeten and pour on the remainder boiling hot; then let it boil slowly for ten minutes in a covered vessel.

These are but some of the uses of this modest, natural product which is deserving of better consideration at the hands of the medical profession.

